# Quality of Life Analysis of HPV-Positive Oropharyngeal Cancer Patients in a Randomized Trial of Reduced-Dose Versus Standard Chemoradiotherapy: 5-Year Follow-Up

**DOI:** 10.3389/fonc.2022.859992

**Published:** 2022-04-08

**Authors:** Mai Takahashi, Michael Hwang, Krysztof Misiukiewicz, Vishal Gupta, Brett A. Miles, Richard Bakst, Eric Genden, Isaiah Selkridge, John Botzler, Vruti Virani, Erin Moshier, Marcelo R. Bonomi, Marshall R. Posner

**Affiliations:** ^1^ The Department of Medicine, Icahn School of Medicine at Mount Sinai, Mount Sinai Beth Israel, New York, NY, United States; ^2^ The Departments of Hematology/Oncology, Johns Hopkins Hospital, Baltimore, MD, United States; ^3^ The Tisch Cancer Institute, Icahn School of Medicine at Mount Sinai, New York, NY, United States; ^4^ The Departments of Hematology/Oncology, Icahn School of Medicine at Mount Sinai, New York, NY, United States; ^5^ Radiation Oncology, Icahn School of Medicine at Mount Sinai, New York, NY, United States; ^6^ Otolaryngology, Icahn School of Medicine at Mount Sinai, New York, NY, United States; ^7^ Biostatistics in the Icahn School of Medicine at Mount Sinai, New York, NY, United States; ^8^ The Departments of Hematology/Oncology, The Ohio State University, Columbus, OH, United States

**Keywords:** head and neck cancer, de-escalation therapy, quality of life analysis, HPV-positive squamous cell carcinoma, chemoradiotherapy (CRT)

## Abstract

**Background:**

Human papillomavirus-positive oropharyngeal carcinoma (HPVOPC) portends a more favorable prognosis compared to environmentally related oropharynx cancer (EROPC). Patients with HPVOPC may be overtreated and endure unnecessary long-term toxicities.

**Methods:**

Patients with untreated locally advanced HPVOPC received induction chemotherapy with docetaxel, cisplatin, and 5-fluorouracil (TPF) and were randomized to standard chemoradiotherapy (sdCRT) (70 Gy) or reduced-dose chemoradiotherapy (rdCRT) (56 Gy) with weekly carboplatin. Patients were followed for changes in five validated quality of life (QoL) surveys: MD Anderson Dysphagia Inventory and Symptom Inventory for head and neck cancer (MDADI, MDASI-HN), Xerostomia Questionnaire (XQ), and European Organization for Research and Treatment of Cancer Questionnaire (EORTC) with head and neck module (EORTC HN). The secondary endpoints of this study were 5-year progression-free survival (PFS) and overall survival (OS).

**Results:**

Twenty patients were enrolled and randomized to rdCRT (n = 12) or sdCRT (n = 8). Median follow-up was 88 months. At 5 years, difference in QoL changes all favored the rdCRT arm and two QoL scales reached statistical significance (EORTC global health score: 11.49 vs. -23.94, P = 0.014; EORTC symptom scale: -7.76 vs. 15.19, P = 0.015). The 5-year PFS and OS were 87.5% and 83.3% for sdCRT and rdCRT, respectively.

**Conclusions:**

Therefore, rdCRT after TPF in HPVOPC is feasible in accordance with the earlier results of the Quarterback Trial and long-term follow-up. These limited results are more favorable in specific QoL domains compared to those of sdCRT and demonstrate equivalent long-term survival.

**Clinical Trial Registration:**

https://clinicaltrials.gov/ct2/show/NCT01706939, The Quarterback Trial [NCT 01706939].

## Introduction

There has been a significant increase in the incidence of oropharynx cancer (OPC) in North America and Europe (1) due to an increase in the incidence of tumors that contain human papillomavirus (HPV), most often HPV16. HPV-positive OPC (HPVOPC) now accounts for more than 60% of OPC seen in the United States and an increasing fraction of these malignancies in Europe (1–3).

HPVOPC has a significantly favorable prognosis compared to environmentally related oropharynx cancer (EROPC) (4–8). Patients with HPVOPC are generally younger, have fewer comorbidities, and have a higher response rate after chemoradiation treatment, although it was recently reported that the incidence of HPVOPC has been increasing among older patients in the United States (9–11). Locoregional control (LRC) in locally advanced HPVOPC is approximately 80%–85% with standard treatment, while LRC in locally advanced non-HPV OPC is approximately 35%–45% (12, 13). Thus, HPVOPC patients are cured at a higher rate and survive longer. As a result, survivors are at high risk (HR) for long-term toxicity and mortality from current therapies. This has prompted trials to de-escalate therapies and improve quality of life (QoL).

Chemoradiotherapy (CRT) increases early and late toxicities compared to radiation therapy alone including increased late mortality as described in RTOG 91-11 (14, 15). The primary modifiable causes of acute and late toxicity from CRT include the addition and type of chemotherapy, radiation therapy dose, and radiation field size (13, 16–19). Acute toxicities from CRT include mucositis, dermatitis, ototoxicity, dysphagia, xerostomia, nausea, vomiting, and pain. Late toxicities include dysphagia, xerostomia, dental failure, aspiration, fibrosis, tissue necrosis, hypothyroidism, and osteoradionecrosis (18, 20, 21).

A variety of de-escalation strategies have emerged, including changes in dose or choice of cytotoxic chemotherapy, dose or volume reductions of radiotherapy, and use of less invasive surgical techniques such as transoral robotic surgery (9, 22–29). A currently investigated strategy involves utilizing response to induction therapy to select patients for de-escalated definitive therapy and to reduce locoregional and distant failure (30, 31). Response to induction therapy has been shown to predict the response to definitive radiotherapy or combined CRT (32, 33). A number of trials have recently been published using induction followed by a risk-adapted locoregional therapy (9, 34–36).

To prevent overtreatment, long-term morbidity, and deterioration in QoL, multiple studies have focused on de-intensification techniques with reduced-dose radiation therapy for HPVOPC treatment (9, 22–26). We report here the long-term results of the Quarterback Trial, directly comparing a reduced-dose chemoradiotherapy (rdCRT) to standard of care (sdCRT) after induction chemotherapy (IC) in locally advanced HPVOPC for patient-reported QoL, toxicity, and survival in the two treatment arms.

## Methods

### Study Design and Participants

The Quarterback Trial (NCT 01706939) is a randomized phase III non-inferiority trial approved by the institutional review board (IRB) of Icahn School of Medicine at Mount Sinai (20). The trial evaluated rdCRT vs. sdCRT in patients with untreated American Joint Commission on Cancer (AJCC) 7th edition stage III or IV HPVOPC without evidence of distant metastases who were entered after signing a consent form. All eligibility criteria for the Quarterback Trial are described in the original publication (20). In brief, patients who were immunohistochemically confirmed p16+ and PCR-positive HR HPV with locally advanced unknown primary or primary cancer of the supraglottic larynx, hypopharynx, nasopharynx, and oropharynx were eligible. A smoking history of ≤20 pack-years was required, and patients could not be active cigarette smokers, defined as at least 1 cigarette per day in the last 5 years. Patients were treated with 3 cycles of TPF IC and then randomized (2:1) to rdCRT at 5,600 cGy or sdCRT at 7,000 cGy, both given with weekly carboplatin at area under the curve (AUC) of 1.5. The first 4 patients were also treated with cetuximab at 400 mg/m^2^ loading dose followed by 250 mg/m^2^ weekly to the end of radiotherapy. Due to an increase in mucositis seen in these 4 patients, the protocol was amended and carboplatin at AUC 1.5 was given alone. No prophylactic percutaneous endoscopic gastrostomy (PEG) tubes were placed. All patients were treated with daily intensity-modulated radiotherapy (IMRT) as described previously.

### Quality of Life Assessment

QoL was assessed prospectively using 5 validated QoL surveys: MD Anderson Dysphagia Inventory (MDADI), MD Anderson Symptom Inventory for head and neck cancer (MDASI-HN) with subscore of symptom interference (SI) and symptom severity (SS), the University of Michigan Xerostomia Questionnaire (XQ), the European Organization for Research and Treatment of Cancer Questionnaire (EORTC) with subscore of global health scale (GHS), functional scale (FS), and symptom scale (SS), and the EORTC supplementary head and neck cancer module (EORTC HN). Additional description of QoL modules is provided in **Supplementary Material 1**.

QoL data were collected at baseline, weekly during IC, every 2 weeks with CRT, and at 3, 6, 12, and 24 months’ and 5 years’ follow-up after completion of CRT by clinical research coordinators. Due to lower compliance at 3 months from both arms (2 patients missing from rdCRT, 3 patients from sdCRT), responses from 3-month and 6-month follow-up were combined to create a 3–6-month follow-up score. The score denoting a worse QoL at either 3 months or 6 months was used for each questionnaire, and the other score (if obtained) was discarded. All changes in QoL score were calculated as variances from each patient’s respective baseline scores.

### Statistical Analysis

As described in the initial paper (20), the original statistical plan was revised due to poor accrual. The trial enrollment was terminated after 20 evaluable patients were randomized.

The QoL analysis population included all patients who completed baseline QoL assessment and did not progress. Compliance rates were calculated as the number of forms received divided by the number of forms expected at each time point. Expected forms were from patients alive at the given time point, regardless of disease state.

All statistical analyses were performed using SAS version 9.4. A mixed-model ANOVA was used to estimate changes from baseline QoL to that at each follow-up time point and to compare the difference in QoL changes between the treatment arms. Progression-free survival (PFS) and overall survival (OS) were assessed using the Kaplan–Meier method with comparison with two-sided log-rank test. A P value <0.05 was considered statistically significant, and all P values were two-sided.

The data cutoff date for final analysis was May 1, 2021.

## Results

### Baseline Characteristics

From December 2012 to February 2016, a total of 23 patients were identified with locally advanced HPVOPC and were screened. Two patients did not meet inclusion criteria and were excluded, and one patient positive for HPV18 withdrew from the study after 2 cycles of IC by personal choice. The remaining 20 patients were randomized: 8 patients received sdCRT and 12 patients received rdCRT.

**Figure 1:**
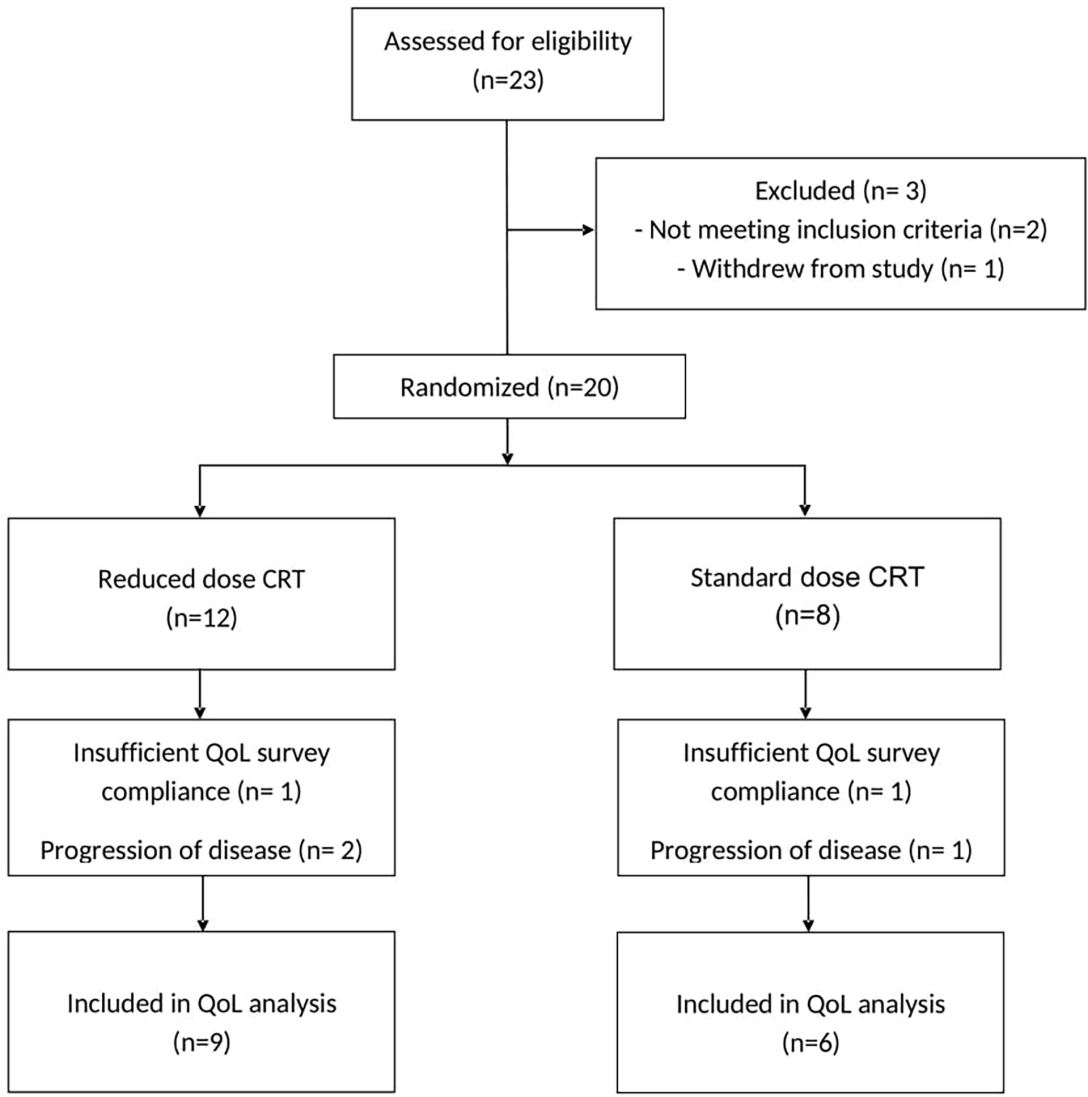
CONSORT Flow Diagram.

All 20 patients were analyzed for survival outcomes. Three-year survival results are reported in a separate manuscript (20). In this study, 70% had HR features including T4, N2c, or N3 (based on AJCC 7th edition) and/or radiographic extracapsular extension (ECE). Four patients who received additional cetuximab with cisplatin and radiation were all assigned to the rdCRT arm. Among the 20 patients, 15 (75%) subjects were included in the final QoL assessment (three patients had progression of disease within the first 4 months after treatment completion, and two patients were excluded due to insufficient survey compliance). Of the remaining 15 patients (75%), 6 were randomized to sdCRT and 9 to rdCRT, as seen in the CONSORT diagram (**Figure 1**). QoL patient characteristics are provided in **Table 1**. Baseline characteristics of the QoL populations were well balanced between the two treatment arms.

**Table 1:** Patient characteristics for QoL subjects.

	rdCRT (N = 9)	sdCRT (N = 6)
**Sex**		
Men	8 (89%)	6 (100%)
Women	1 (11%)	0 (0%)
**Age (years)**		
Mean	61.89	60
Range	52–82	55–64
**Tumor site**		
BOT	5 (55.6%)	4 (66.7%)
Tonsil	4 (44.4%)	2 (33.3%)
**HPV status**		
16	9 (100%)	5 (83.4%)
Other	0 (0%)	1 (16.7%)
**Smoking status**		
Never Smoker	6 (66.7%)	1 (16.7%)
<10 pack-years	2 (22.2%)	3 (50%)
10–20 pack-years	1 (11.1%)	2 (33.3%)
**Race**		
White	5 (55.6%)	4 (66.7%)
African-American	4 (44.4%)	2 (33.3%)

QoL, quality of life; rdCRT, reduced-dose chemoradiotherapy; sdCRT, standard-dose chemoradiotherapy; HPV, human papillomavirus; BOT, base of tongue.

### Quality of Life Analysis

Compliance to the questionnaires was 100% at baseline, 97% during IC, 93% at end of CRT, 73% at 3 months, 93% at 6 months, 40% at 12 months, 53% at 24 months, and 67% at 5 years. Compliance was not statistically different in both arms at all points in time.

As shown in **Supplementary Table S1**, all baseline QoL scores were not significantly different between rdCRT and sdCRT groups; MDADI (74.33 vs. 86.83, P = 0.21), XQ (2.64 vs. 0.79, P = 0.12), MDASI-HN SI (2.78 vs. 0.1, P = 0.18), MDASI-HN SS (2.13 vs. 0.92, P = 0.23), EORTC GHS (66.67 vs. 84.72, P = 0.14), EORTC FS (77.20 vs. 88.33, P = 0.10), EORTC SS (22.63 vs. 14.10, P = 0.27), and EORTC HN (25.08 vs. 8.68, P = 0.07).

At the end of CRT, multiple scales showed significantly less decrement in QoL in the rdCRT arm, as seen in **Table 2**: the EORTC GHS (-14.81 vs. -40.70, P = 0.04), EORTC FS (-9.98 vs. -32.55, P = 0.01), EORTC SS (14.06 vs. 32.11, P = 0.02), and EORTC HN (20.18 vs. 35.69, P = 0.047). The remaining questionnaires did not reveal any significant difference.

**Table 2:** Variance from baseline score.

	After CRT			3–6 Months			12 Months			24 Months			5 Years		
	rdCRT	sdCRT	P	rdCRT	sdCRT	P	rdCRT	sdCRT	P	rdCRT	sdCRT	P	rdCRT	sdCRT	P
**MDADI** ^a^	-27.82	-34.98	0.45	-10.44	-37.53	**0.01**	5.4	-17.13	0.11	-6.29	-8.29	0.88	-0.75	-11.76	0.37
**XQ** ^b^	4.01	5.16	0.42	2.99	5.47	0.09	1.18	2.98	0.41	1.31	3.44	0.29	1.55	4.69	0.10
**DASI-HN SI** ^b^	2.65	4.69	0.15	-0.22	4.07	**0.004**	-1.83	0.99	0.20	-0.88	0.34	0.55	-0.45	1.36	0.34
**MDASI-HN SS** ^b^	3.23	3.84	0.58	0.84	2.90	0.07	-0.74	0.57	0.32	-0.19	1.26	0.23	0.06	1.57	0.18
**EORTC GHS** ^a^	-14.81	-40.70	**0.04**	1.85	-33.24	**0.008**	15.23	-10.58	0.11	7.17	-1.78	0.55	11.49	-23.94	**0.01**
**EORTC FS** ^a^	-9.98	-32.55	**0.01**	3.51	-24.35	**0.002**	11.85	-11.73	0.08	7.26	-6.16	0.28	9.35	-8.16	0.13
**EORTC SS** ^b^	14.06	32.11	**0.02**	0.48	14.16	0.08	-14.37	4.48	0.08	-12.64	0.87	0.17	-7.76	15.19	**0.01**
**EORTC HN** ^b^	20.18	35.69	**0.047**	0.23	23.56	**0.004**	-10.4	11.06	**0.03**	-9.52	14.74	**0.01**	-7.49	7.90	0.061

rdCRT, reduced-dose chemoradiotherapy; sdCRT, standard-dose chemoradiotherapy; IC, induction chemotherapy; CRT, chemoradiotherapy; MDADI, MD Anderson Dysphagia Inventory; XQ, Xerostomia Questionnaire; MDASI SI and SS, MD Anderson Symptom Inventory Symptom Inventory and Severity; EORTC GHS, FS, SS, HN, European Organization for Research and Treatment of Cancer Questionnaire Global Health Scale, Functional Scale, Symptom Scale, and Head and Neck Module.

a A higher or positive score reflects a better quality of life when compared to baseline.

b A lower or negative score reflects a better quality of life when compared to baseline. P values with statistical significance are bolded.

At 3–6-month follow-up, patients in the rdCRT arm had significant QoL recovery compared to sdCRT, as measured by MDADI, MDASI-HN SI, EORTC subscores of GHS and FS, and EORTC HN supplement (-10.44 vs. -37.53, P = 0.01; -0.22 vs. 4.07, P = 0.004; 1.85 vs. -33.24, P = 0.008; 3.51 vs. -24.35, P = 0.002; 0.23 vs. 23.56, P = 0.004, respectively). The difference in XQ, MDASI-HN SS, and EORTC SS showed a nearly significant trend (2.99 vs. 5.47, P = 0.09; 0.84 vs. 2.90, P = 0.07; 0.48 vs. 14.16, P = 0.08).

At 12 months and 24 months posttreatment, there was no significant difference in QoL changes between both arms except EORTC HN (-10.4 vs. 11.06, P = 0.025; -9.52 vs. 14.74, P = 0.007). QoL scores had returned to near their baselines established prior to CRT. At 5 years’ follow-up, differences in QoL changes all favored the rdCRT arm and EORTC GHS and EORTC SS reached statistical significance (11.49 vs. -23.94, P = 0.014; -7.76 vs. 15.19, P = 0.015). The difference in QoL by EORTC HN approached significance (-7.49 vs. 7.90, P = 0.061).

### PEG Tube Placement

Two of 15 patients required PEG tube: 1 in the rdCRT arm who received cetuximab and carboplatin and 1 patient in the sdCRT arm receiving carboplatin only. There was no significant difference in baseline QoL scores or QoL scores at last CRT session in all surveys between patients with PEGs placed and patients without. Patients with PEGs placed, however, did have a larger decrement in QoL, as scored by the MDADI questionnaire, which approached significance (-26.37 vs. -50.5, P = 0.056). All PEGs were removed by 8 months after CRT.

### Progression-Free and Overall Survival

**Figure 2:**
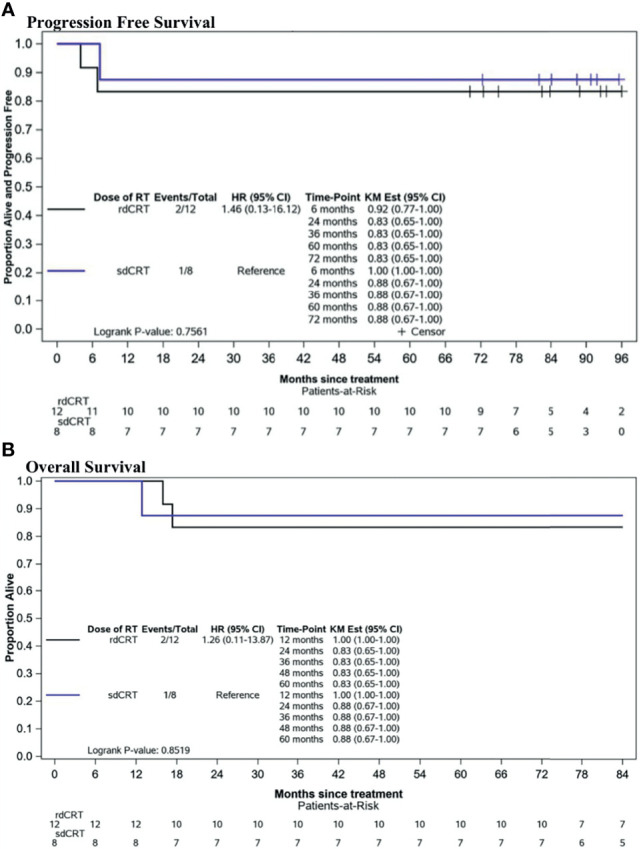
Kaplan Meier Plots for Progression Free Survival and Overall Survival. **(A)** Progression Free Survival. **(B)** Overall Survival.

As of May 1, 2021, surviving patients have been followed for a median of 88 months (range, 72–100). All patients who progressed did so within the first 12 months after treatment start. The 5-year PFS and OS are identical for each cohort and are 87.5% vs. 83.3% (P = 0.76, P = 0.85) for sdCRT and rdCRT, respectively (**Figures 2A, B**).

### Treatment Toxicity

Pattern of toxicity is listed in **Supplementary Table S2**. Among the 20 patients, 4 patients developed severe adverse events including one in the sdCRT group with an HPV-negative, p16-positive, p53-mutated retromolar trigone oropharyngeal squamous cell carcinoma within the radiation field 7 years after primary therapy; one from the sdCRT arm with osteoradionecrosis; one from the rdCRT arm with dental failure resulting in removal of all teeth; and one from the rdCRT group with severe oropharyngeal scarring and fibrosis. This last patient had preexisting systemic lupus erythematosus, a risk factor for increased radiation toxicity. Six patients (50%) from the rdCRT arm and three patients (37.5%) from the sdCRT arm had newly diagnosed hypothyroidism posttreatment.

## Discussion

In the Quarterback Trial, rdCRT after IC demonstrated comparable 5-year PFS/OS compared to sdCRT. The role of IC has been discussed in many studies (37–40). Treatment with IC plus CRT has been associated with improved survival in patients with head and neck squamous cell carcinoma (37, 38). We selected the TPF regimen based on the TAX 324 trial that reported a significant benefit and better locoregional disease control in patients who received TPF followed by CRT compared to PF when both were delivered with CRT (37).

In this study, patients with locally advanced HPVOPC receiving rdCRT were already reporting superior QoL as measured by EORTC questionnaire compared to sdCRT at the end of CRT. By 3–6 months posttreatment, patients receiving rdCRT exhibited significantly better QoL in almost all of the validated QoL measurement tools used for testing when compared to patients treated with sdCRT. At 12 months, decrements in QoL resolved and remained at the same level at 24 months’ follow-up. These findings were consistent with previous studies that showed QoL improvements in CRT-treated patients by 12 months (41–43). Although one questionnaire (EORTC HN) showed a significant difference in score both at 12 and 24 months’ follow-up, other modules did not show a statistically significant difference in negative impact on QoL of sdCRT. This is noteworthy since radiation toxicity is typically correlated to total radiation dose. However, at 5 years, differences in QoL changes all favored the rdCRT arm with particular respect to significant benefit in scores on the EORTC GHS and EORTC SS. Our results suggest that although rdCRT was not superior in terms of intermediate-term QoL, longer-term evaluation where radiation toxicity increases over time demonstrated less decline in QoL compared to that in the sdCRT group. Notably, for patients treated with rdCRT, significantly greater improvements in QoL with EORTC HN was observed at almost all follow-up time points. These data indicate that rdCRT is beneficial particularly in terms of maintaining early and late disease-specific QoL and physical functioning. Compliance with completion of QoL questionnaires was high in both arms at baseline, though completion rates were lower at 3 months and 6 months of follow-up, prompting the two QoL scores at these time points to be combined. In addition, although completion rates were lower at 12 and 24 months of follow-up, the 5 years’ follow-up rate reached 67%.

An overall low rate of PEG tube dependence could be explained by less impact of local toxicity on swallowing function and nutrition in this patient population with less smoking history and few comorbidities. IC improves function by tumor reduction prior to radiation and helps to decrease radiation field size. Necessity of PEG tube placement was not significantly different between the two arms. The decrease in QoL after CRT, as quantified by the MDADI questionnaire, however, appears to be correlated with the necessity of a PEG. Although it did not reach statistical significance, this may be due to the small sample size. MDADI score has been previously analyzed in studies comparing prophylactic vs. non-prophylactic PEG tubes. Data from these studies, however, appear to be divided, with one study showing improved MDADI scores in patients with prophylactic PEG tubes (44), while another showed no significant difference (45). Our results suggest that MDADI may be useful in predicting which patients will require a PEG placement during CRT.

The 4 patients who received additional cetuximab were all allocated to the rdCRT group. Previous studies suggest that cetuximab may be effective as a radiation sensitizer with survival advantages; however, the RTOG 0522 trial demonstrated that CRT plus cetuximab did not improve survival outcomes compared to standard CRT (13, 46). Although a higher incidence of acute toxicities with combining cetuximab and standard CRT was observed in RTOG 0522, QoL changes at the end of CRT still overall favored the rdCRT arm in our study.

Two recently published phase II trials reported superior QoL in patients receiving rdCRT in HPVOPC. One study used the University of Washington Quality of Life Questionnaire and Functional Assessment of Cancer Therapy Head and Neck Questionnaire (43), and the other used the EORTC questionnaire and patient-reported outcome version of Common Terminology Criteria for Adverse Events (CTCAE) (47). The study by Chera et al. (47) differs from our study in being nonrandomized and selecting patients with low disease volume and surgery after CRT in those with positive lymph node involvement. The present report supports the notion that rdCRT as part of a sequential therapy program in LA HPVOPC with poor prognostic features is feasible and results in significantly better and quicker improvements in QoL through 12 months post CRT when compared to sdCRT. In this study, greater improvements in QoL at 5 years’ follow-up were reported in the rdCRT group.

It is notable that one patient from the sdCRT arm had a second primary. This was an HPV-negative, p16-positive, p53-mutated, retromolar trigone oropharyngeal squamous cell carcinoma in the radiation field 7 years after the therapy. A robust and more informative evaluation of late effects from CRT is essential and will require a larger population and decades of follow-up. Additionally, superior survival (85% at 5 years) in HPVOPC compared to that expected in advanced non-HPV patients (40%) makes de-escalation of radiation dose a priority question to be addressed in randomized trials. While this study is limited by the very small population studied, even with small numbers, a significant degree of impact in both acute and late effects of de-escalation can be identified, supporting larger randomized trials of this treatment paradigm in advanced patients. As a result of the outcome of this trial, the Quarterback study has been extended as a Phase 2 trial with comparable PFS and OS (48).

Some strengths of our study include its randomization design, long-term follow-up period, and the use of standard and validated questionnaires that allow for direct comparison with other studies. While this is a small study, it is noteworthy that even with small numbers, significant differences were identified acutely and at 5 years of follow-up in the de-escalated arm compared to sdCRT and survival was excellent. Limitations of this study include its small patient sample, patient survey compliance, and possible reporting bias. As is common with QoL evaluations, insufficient compliance limits the interpretation of the results. A lack of completion of surveys at 3 months’ follow-up required 3-month and 6-month QoL scores to be combined to create a 3–6-month follow-up score for each patient. In addition, due to the nature of this study, patients were not blinded and thus may have introduced further reporting bias when they completed their surveys.

In summary, these data support the conclusion that rdCRT after IC with TPF in locally advanced HPVOPC results in greater acute and long-term improvements in QoL when compared to standard care while maintaining survival. This supports the notion that rdCRT after response stratification to IC in LA HPVOPC is worthy of cooperative group randomized trials (49).

## Data Availability Statement

The raw data supporting the conclusions of this article will be made available by the authors without undue reservation.

## Ethics Statement

The studies involving human participants were reviewed and approved by the IRB of Icahn School of Medicine at Mount Sinai. The patients/participants provided their written informed consent to participate in this study.

## Author Contributions

KM, VG, BAM, RB, EG, MRB, and MRP contributed to the conception and design of the study. MT, MH, IS, JB, VV, and MRP organized the database. EM performed the statistical analysis. MT and MH wrote the first draft of the article. All authors contributed to article revision and read and approved the submitted version.

## Funding

Research reported in this publication was supported in part by the National Cancer Institute Cancer Center Support Grant P30CA196521-01 awarded to the Tisch Cancer Institute of the Icahn School of Medicine at Mount Sinai and used the Biostatistics Shared Resource Facility. The content is solely the responsibility of the authors and does not necessarily represent the official views of the National Institutes of Health.

## Conflict of Interest

The authors declare that the research was conducted in the absence of any commercial or financial relationships that could be construed as a potential conflict of interest.

## Publisher’s Note

All claims expressed in this article are solely those of the authors and do not necessarily represent those of their affiliated organizations, or those of the publisher, the editors and the reviewers. Any product that may be evaluated in this article, or claim that may be made by its manufacturer, is not guaranteed or endorsed by the publisher.
