# Supplementary material for: Quality of Life Analysis of HPV-Positive Oropharyngeal Cancer Patients in a Randomized Trial of Reduced-Dose Versus Standard Chemoradiotherapy: 5-Year Follow-Up
Source: Front Oncol. 2022 Apr 8;12:859992. doi: 10.3389/fonc.2022.859992 (PMC9024140; doi:10.3389/fonc.2022.859992)
Supplement: Supplementary file 1 [file DataSheet_1.docx]

**Supplementary Material 1**

Detailed Description Quality of Life Questionnaires and modules:

**MDADI** is a questionnaire of 20 questions and contains a global subscale, and three other categories of questions (emotional, functional, and physical). The scores are summed and a mean score is calculated. This mean score was multiplied by 20 to obtain a score, with a range of 0 (extremely low functioning) to 100 (high functioning). Thus, a higher MDADI score represented better day-to-day functioning and better QOL.

**MDASI Head and Neck** is a site-specific MDASI module which includes the core MDASI 13 symptom severity items (SS) and 6 symptom interference items (SI), alongside 9 items relevant to head and neck cancer. The scores are summed and a mean score is calculated on a scale of 0 (low severity or interference) to 10 (high severity or complete interference). In order to calculate the mean score, a majority of the subscale’s items must have been completed.

**XQ** is a nine questions survey developed specifically for xerostomia symptoms. The scores are summed and a mean score is calculated on a scale of 0 (low xerostomia interference) to 10 (high xerostomia interference).

**The EORTC QLQ-C30** contains a global QoL score, five functional scales (physical, role, cognitive, emotional, and social), three symptom scales (fatigue, nausea/vomiting, and pain), and six single-item scales (dyspnea, sleep disturbance, appetite loss, constipation, diarrhea, and financial difficulties). The EORTC Head and Neck module was specifically designed and validated for head and neck cancer patients. This 35-item questionnaire contains seven symptom scales (pain, swallowing, senses, speech, social eating, social contact, and sexuality), six single-item scales (difficulties of teeth, mouth opening, dry mouth, sticky saliva, coughing, and feeling ill), and five items about the additional use of pain medicine, nutritional supplements, and feeding tube and changes in body weight. All items were transformed to scales from 0 to 100, according to the EORTC scoring manual recommendations, and divided into respective sub-scores of global health (GHS), functional (FS), and symptom scale (SS). A high score on global health and functional scale represents a better level of functioning, whereas a high score on a symptom scale and head and neck module indicates more severe symptoms.

**Supplementary Table 1: Baseline QoL Scores prior to IC**

|  | **rdCRT** | **sdCRT** | ***P Value*** |
| --- | --- | --- | --- |
| **MDADI**^a^ | 74.33 | 86.83 | 0.21 |
| **XQ**^b^ | 2.64 | 0.79 | 0.12 |
| **MDASI-HN SI**^b^ | 2.78 | 0.1 | 0.18 |
| **MDASI-HN SS**^b^ | 2.13 | 0.92 | 0.23 |
| **EORTC GHS**^a^ | 66.67 | 84.72 | 0.14 |
| **EORTC FS**^a^ | 72.20 | 88.33 | 0.10 |
| **EORTC SS**^b^ | 22.63 | 14.10 | 0.27 |
| **EORTC HN**^b^ | 25.08 | 8.68 | 0.07 |

QoL: Quality of Life, IC: Induction Chemotherapy, rdCRT: Reduced Dose Chemoradiotherapy, sdCRT: Standard Dose Chemoradiotherapy, IC: Induction Chemotherapy, CRT: Chemoradiotherapy, MDADI: MD Anderson Dysphagia Inventory, XQ: Xerostomia Questionnaire, MDASI SI and SS: MD Anderson Symptom Inventory Symptom Inventory and Severity, EORTC GHS, FS, SS, HN: European Organization for Research and Treatment of Cancer Questionnaire Global Health Scale, Functional Scale, Symptom Scale, and Head and Neck Module

^a^A higher or positive score reflects a better quality of life when compared to baseline

^b^A lower or negative score reflects a better quality of life when compared to baseline

P values with statistical significance are italicized.

**Supplementary Table 2: Adverse Events**

| Patient | Arm | Age | Sex | Stage | Toxicity/Event |
| --- | --- | --- | --- | --- | --- |
| 1 | rdCRT | 62 | M | IV (T3, N2b, M0) | Dental failure with removal of all teeth |
| 2 | rdCRT | 67 | M | IVa (T2, N2b, M0) | Severe oropharyngeal scarring and fibrosis |
| 3 | sdCRT | 67 | M | IVa (Tx, N2c, M0) | Second primary disease |
| 4 | sdCRT | 73 | M | IV (T4a, N0, M0) | Osteoradionecrosis |

rdCRT: Reduced Dose Chemoradiotherapy, sdCRT: Standard Dose Chemoradiotherapy
